# Trichlorophenate of Lime

**Published:** 1883-11

**Authors:** 


### Trichlorophenate of Lime.

The *Med. Press and Circular*, July 18, 1883, says:

A Russian medical journal publishes an account of experiments made by one of its collaborators on the antiseptic properties of trichlorophenate of lime, a mixture of phenic acid and chloride of calcium. From this it appears that this compound is twenty times stronger as an antiseptic than phenic acid, permanganate of potassa, chloride of calcium, salicylic acid, or boric acid. It is not only disinfectant, but removes all odors from wounds. It is essentially useful in soft chancre, diphtheria, and other gangrenous affections. Trichlorate of phenol is not only easily and quickly prepared, but its price is also less than that of phenic acid.

Amen!—We take it that there is too universal a belief in the homœopathic law, too general a use of it in the practice of homœopathic physicians, too sure a knowledge that the only way to propagate a truth distasteful to its opponents is by fighting with a banner, too honest a faith that, when homœopathy has been fully developed and simplified, an ordinary man will seldom need to go to any other resource, for us just now to forsake the name which is compelling the world to listen to the truths of scientific therapeutics. Not, we think, till this war is over, will the homœopathist be ready to give up that by which he is known, and for which the world respects him.—*Homœopathic Leader.*

"There is nothing very brilliant about our Bremen dentists," said a lady to a member of the profession in Berlin, "but they are very obliging. If you wish a tooth extracted with gas, they forthwith light the chandelier."

In distributing the prizes awarded to the successful students at the Macclesfield Grammar School, England, the Bishop of Manchester condemned the system of "cramming," which he said was too common in our schools. The Americans had found it to be a false educational basis, and had to retrace their steps.

